# Tracheal laceration following rapid sequence intubation

**DOI:** 10.36416/1806-3756/e20240174

**Published:** 2024-08-07

**Authors:** Filipa Jesus, Élin Almeida, Alcina Tavares

**Affiliations:** 1. Serviço de Pneumologia, Unidade Local de Saúde da Guarda EPE, Guarda, Portugal.; 2. Faculdade de Ciências da Saúde, Universidade da Beira Interior, Covilhã, Portugal.

**Figure 1:**
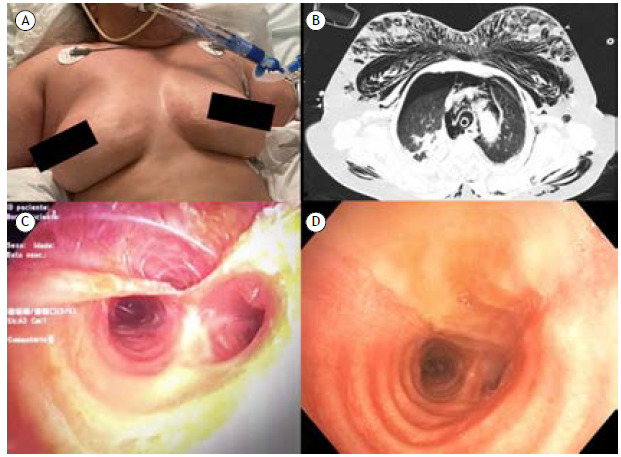
In A, a patient with significant subcutaneous emphysema after being initiated on invasive mechanical ventilation. In B, a chest CT scan revealed exuberant subcutaneous emphysema, pneumomediastinum, and bilateral pneumothorax. In C, a flexible bronchoscopy was performed after intubation, and the image shows a laceration on the lower third of the posterior tracheal wall (anterior view). In D, a repeat flexible bronchoscopy was performed 12 days after the initial endoscopic evaluation, and the image shows complete reepithelization of the posterior tracheal wall (anterior view).

A 64-year-old female with major depressive disorder was admitted to the ER after voluntary intoxication with amitriptyline, venlafaxine, and lamotrigine. Upon admission, a Glasgow Coma Scale of 3 was documented, and rapid sequence intubation was promptly performed, initiating the patient on invasive mechanical ventilation. After being transferred to the ICU, subcutaneous emphysema was noted (Figure 1A). Chest CT showed exuberant pneumomediastinum and bilateral pneumothorax (Figure 1B). After chest tube placement, flexible bronchoscopy was performed (Olympus® BF-H190, Olympus, Japan) showing a laceration on the lower third of the posterior tracheal wall (Figure 1C). A double-lumen tube was used to replace the previous single-lumen endotracheal tube, allowing adequate ventilation while bypassing the damaged area and allowing cicatrization. Twelve days after the procedure, endoscopic reassessment (Olympus® BF-H190) showed complete reepithelization of that injury (Figure 1D).

Post-intubation tracheal laceration is a rare but a potentially life-threatening condition, with an overall incidence of 1 per 20.000, increasing up to 15% following emergency intubation.^1^ Intubation injuries are more common in females, probably due to a shorter average tracheal length and weaker *pars membranosa.* Subcutaneous emphysema is the most common symptom and also a protective factor, as it favors early diagnosis and rapid initiation of appropriate treatment.^2^
